# Changes in Water Sorption and Solubility of Dental Adhesive Systems after Cigarette Smoke

**DOI:** 10.1155/2013/605847

**Published:** 2013-07-28

**Authors:** Lívia Andrade Vitória, Thaiane Rodrigues Aguiar, Poliana Ramos Braga Santos, Andrea Nóbrega Cavalcanti, Paula Mathias

**Affiliations:** ^1^Department of Clinical Dentistry, School of Dentistry, Federal University of Bahia, Av. Araújo Pinho, 62, Canela, 40110-150 Salvador, BA, Brazil; ^2^Department of Restorative Dentistry, College of Dentistry, University of Illinois at Chicago, 801 South Paulina Street, Chicago, IL 60612, USA

## Abstract

*Aim*. To evaluate the effect of cigarette smoke on water sorption and solubility of four adhesive systems. *Materials and Methods*. Sixteen disks of each adhesive system were prepared (Adper Scotchbond Multipurpose Adhesive (SA); Adper Scotchbond Multipurpose Adhesive System (Adhesive + Primer) (SAP); Adper Single Bond Plus (SB); Adper Easy One (EO)). Specimens were desiccated until a constant mass was obtained and divided into two groups (*n* = 8). One-half of the specimens were immersed in deionized water, while the other half were also immersed, but with daily exposure to tobacco smoke. After 21 days, disks were measured again and stored in desiccators until constant mass was achieved. Data were calculated according to ISO specifications and statistically analyzed. *Results*. The tobacco smoke only significantly affected the water sorption and solubility of EO. There were significant differences in both analyses among materials tested. The SB exhibited the highest water sorption, followed by EO, which demonstrated significantly higher solubility values than SB. The SA and SAP showed low water sorption and solubility, and there were no significant differences between the two. *Conclusion*. Regardless of smoke exposure, both simplified adhesive systems presented an inferior performance that could be related to the complex mixture of components in such versions.

## 1. Introduction

Many studies have shown that cigarette smoking is associated with deleterious health effects such as heart disease [1], chronic obstructive pulmonary disease, cancer [2], fertility problems [3], and periodontal disease [4]. In cosmetic restorative dentistry, some of the substances coming from tobacco can be absorbed by resin composite, dentin, and enamel surface [5] and lead to tooth/composite discoloration [6–10]. Moreover, bond strength reduction has been described due to the fact that cigarette particles may prevent effective contact between dentin and resin composite [11].

The mainstream smoke emitted from the mouth end is complex and composed by many physical and chemical processes [12]. In general, smoke formation is mostly produced by combustion and pyrolysis reactions and can be classified in two distinct phases. The first is called the vapour phase, consisting mainly of nitrogen, oxygen, carbon monoxide, carbon dioxide, acetaldehyde, methane, hydrogen cyanide, nitric acid, and acetone. The second category is the particulate phase. It consists of nicotine, water, and tobacco-specific nitrosamines ranging from 0.1 to 1.0 *μ*m in diameter [12–17].

Beside many research reports on the staining effect of tobacco smoke on resin-based materials [6–9] and the positive correlation between tooth discoloration and duration-frequency of smoking habit [10], detailed information is not yet available concerning the effects of tobacco exposure on the physical and mechanical properties of dental restorative materials. Therefore, understanding the effects of cigarette smoke using a burning cigarette in vitro method can be helpful to predict the impact of cigarette smoking on the oral environment and on the long term of dental restorations.

In addition, water absorbed into the resin-based material might contribute to an accelerated degradation, promoting the deterioration of polymer resin [18–20]. The negative aspect of this mechanism includes softening of dental resin through plasticization, filler debonding, and residual monomer release of resin matrix that results in weight loss and characterizes the solubility behavior [19, 21]. Furthermore, water sorption and solubility can affect other properties (i.e., flexural and compressive strength and roughness [22, 23]) and have an important value in terms of the durability of restoration procedures.

Previous studies of water sorption and solubility have mainly been conducted in water [18, 19, 22, 24], artificial saliva [25], lactic acid [26], different pH beverages [23], ethanol, and chloroform [27]. To date, the ability of volatile compounds released during the cigarette combustion to affect the water sorption and solubility of resin-based materials is not yet described. Thus, the aim of the present study was to analyze whether cigarette smoke affects the water sorption and solubility of different adhesive systems. The null hypotheses tested were that (1) cigarette smoke has no effect on the water sorption and solubility of adhesive systems and, (2) none of the adhesive systems tested would have different performance.

## 2. Materials and Methods

### 2.1. Experimental Design

Four adhesive systems were investigated in this study: two conventional adhesive systems (Adper Scotchbond Multipurpose Adhesive and Adper Scotchbond Multipurpose System (Primer + Adhesive)); one two-step etch-and-rinse system (Adper Single Bond Plus) and one self-etching adhesive (Adper Easy One). Their composition specifications, manufacturer, and batch numbers are listed in Table 1.

### 2.2. Specimens Preparation

The sorption and solubility test was performed based on the ISO 4049 standard specification, except for the period of water immersion and cigarette smoke treatment. Sixteen disk-shaped specimens of each adhesive system were prepared (15.0 ± 0.1 mm in diameter ×1.0 ± 0.1 mm depth). The adhesive was directly applied to the metal mold, and a 60-second dwell time was used for solvent evaporation [28], followed by two seconds being gently blown with an oil/water-free compressed air at a distance of 10 cm. The specimens were light-cured for 15 seconds on the middle of the sample, and additional polymerization was applied at four equally peripheral points on the circumference lasting 10 seconds each, resulting in a total curing time of 55 seconds. The light-emitting diode curing unit (Radii-cal, SDI Dental Product, Bayswater Victoria, Australia) was used with a light power density of 1.200 mW/cm^2^. The output irradiance was measured with a radiometer unit (Gnatus, Ribeirão Preto, SP, Brazil).

### 2.3. Water Sorption/Solubility and Smoke Exposure

Immediately after polymerization, all specimens were stored in a desiccator filled with anhydrous calcium sulphate (CaSO_4_) at 37°C for 24 hours. Specimens were weighed using an electronic balance (AUD 220D, Shimadzu Corp., Nakagyo-ku, Kyoto, Japan) to an accuracy of ±0.1 mg. This procedure was repeated until a constant mass was obtained (*M*1), that is, until the mass loss of each specimen was not more than 0.1 mg within a period of 24 hours. The diameter and thickness of each specimen were measured using a digital electronic caliper (Mitutoyo Sul Americana Ltda, São Paulo, SP, Brazil), rounded to the nearest 0.01 mm. These measurements were taken in order to calculate the volume of each specimen in mm^3^.

Half of the specimens (*n* = 8) were placed into polyethylene vials containing 5 mL of deionized water and individually stored for 21 days, while the other half was exposed to tobacco smoke and deionized water. For tobacco smoke-water treatment, specimens were daily exposed to tobacco smoke (10 cigarettes per 8 minutes, twice a day) [8, 9], washed and stored in deionized water after each cycle of smoke exposure for 21 days. The deionized water was renewed daily for all groups.

The cigarettes used presented an elevated content of tar (10 mg; Hollywood Original Blend, Souza Cruz SA, São Paulo, Brazil). The method used in this study was described in previous reports [8, 9, 29]. The tobacco smoke apparatus consisted of two chambers connected by orifices with cigarette filter papers, hermetically sealed. Lit cigarettes were placed in the first chamber that received external ventilation from an air pump, providing constant airflow. The specimens were placed in the second chamber where smoke-air was drawn through from the first chamber and had to overcome the tar filter barrier. In the second chamber, there was a special orifice to release the air stream.

 After 21 days, specimens had excess water gently wiped off with absorbent paper and weighed (*M*2). Then, disks were conditioned again in the same manner as described before until a constant weight was achieved (*M*3). The values of water sorption (WS) and solubility (SL) were calculated as

(1) $$\begin{array}{c} \text{WS}=\frac{M2-M3}{V},\\ \text{SL}=\frac{M1-M3}{V}, \end{array}$$

where *M*1 is the initial dry constant mass (*μ*g) prior to immersion in water; *M*2 is the mass of the specimen (*μ*g) after immersion in water or submitted to smoke treatment/water immersion for 21 days; *M*3 is the mass of the reconditioned specimen (*μ*g), and *V* is the specimen volume in mm^3^.

### 2.4. Statistical Analysis

Water sorption and solubility were expressed in *μ*g/mm^3^ and the data were analyzed by two-way ANOVA and Tukey tests. Statistical analysis was carried out in the SAS 9.1 statistical package (SAS Institute, Cary, NC, USA) with a 95% confidence level.

## 3. Results

The results of the experimental groups are presented in Table 2. A statistically significant interaction between adhesive system and cigarette smoke condition was observed for water sorption (*P* = 0.04) and solubility (*P* = 0.01). The cigarette smoke significantly increased the water sorption and solubility of the self-etching adhesive system (Adper Easy One). 

Regardless of the experimental condition, the adhesive Adper Single Bond Plus showed significantly higher water sorption values than all the other materials. On the other hand, the Adper Easy One exhibited higher solubility than Adper Single Bond Plus. The Adper Scotchbond Multipurpose Adhesive and Adper Scotchbond Multipurpose Systems (Adhesive + Primer) exhibited the lowest water sorption and solubility values, and there were no significant differences between them.

## 4. Discussion

Cigarette smoke is a dynamic mixture of at least 5600 chemicals and toxicants compounds. Some investigations have focused on the identification and quantifying of the particulate phase and volatile smoke components [12, 16, 17]. According to Bartalis et al. [17], the radicals detected in cigarette smoke are reactive and may promote oxidation; however, little is known about the effect of smoke cigarette on the physical and mechanical properties of resin-based materials. In this study, smoke exposure (10 cigarettes—8 minutes/twice a day/21 days) led to an increase of water sorption (12.5%) and solubility (7.9%) of Adper Easy One self-etching adhesive. Therefore, the first hypothesis was rejected since cigarette smoke significantly affected the water sorption and solubility of the self-etching adhesive system.

Most current adhesive systems are based on hydrophilic monomers for bonding to wet dentin substrate. The chemical composition of resin-based materials (i.e., hydrophilic and hydrophobic monomers, solvent content, and filler particles) is deemed to play crucial role in water sorption/solubility [18, 19, 21, 24, 30–32]. In the present study, the higher water sorption values were obtained for two-step etch-and-rinse Adper Single Bond Plus (268.5 and 275.1 *μ*g/mm^3^), followed by self-etching Adper Easy One (216.5 and 243.6 *μ*g/mm^3^) from two groups studied (control and cigarette smoke, resp.). Since changes in water sorption and solubility among adhesive systems were statistically observed, the second hypothesis was rejected.

In agreement with others studies, simplified adhesive systems have more acidity by increasing the concentration of ionic and/or acidic monomers [19, 21] and hence may result in more water uptake and solubility as showed in this investigation. Differences in amount of solvent and monomers constituents may lead to higher water sorption for Adper Single Bond Plus than for Adper Easy One.

On the other hand, conventional adhesive systems (Adper Scotchbond Multipurpose Adhesive and Primer/Adhesive) exhibited the lower water sorption, and there were no significant differences between them. The results indicated that hydrophobic resin coating (Adper Scotchbond Multipurpose Adhesive) has similar behavior when it was used alone or with additional hydrophilic primer (Adper Scotchbond Multipurpose Primer). Thus, these findings seem to indicate that additional hydrophobic resin coating can improve performance of dental adhesive.

In regard to solubility behavior, Adper Easy One demonstrated significantly higher values than Adper Single Bond Plus, followed by conventional systems. According to Ito et al. [18], higher water sorption value may be related to higher solubility. The high concentration of hydrophilic monomers in simplified adhesives can affect the vapor pressure of volatile components [30, 33]. Thus, after polymerization, nanopores may be present due to residual solvent evaporation and hence increase the water uptake. In addition, the simplified etch-and-rinse adhesive showed better ethanol solvent evaporation and monomer infiltration using 60-second dwell times [28]. Solvent evaporation was performed in this study; however, 60-second dwell time plus 2 seconds gently blown at a distance of 10 cm may not be sufficient to remove most of the water/ethanol for all tested adhesive systems. It can be speculated that solvent present in simplified systems may still remain in the adhesive and tends to promote more water sorption and solubility than in conventional systems.

The solubility results of Adper Scotchbond Multipurpose Adhesive and Primer/Adhesive did not show any significant differences. Two possible explanations for the negative solubility values are assumed. The first one was that the absorbed water during storage period may have not been completely eliminated, and consequently mass gain was obtained [27, 30, 32]. Secondly, the volatile smoke compounds may be linked to the adhesive systems specimens and led to increase the mass values.

It is well established that resin-based materials change color when exposed to cigarette smoke [7–9], and smoke staining agents were not removed after composite resin repolishing procedures [7]. Although the chemical mechanisms between cigarette staining and resin-based materials are not clear yet, cigarette-staining agents likely interacted with resin composite that is not only superficial. Thus, it is supposed that this interaction may be extended to adhesives systems due to the fact that adhesive systems have a greater amount of organic matrix than resin composite. In addition, high temperature exposure increases the rate of diffusion and the water sorption/solubility of dental adhesive, resulting in an irreversible degradation of the polymer network [32]. These results may be due to short-term effects of cigarette smoke and water storage used in this investigation or even to the fact that the temperatures reached by lit cigarettes were not high enough to provide matrix chemical degradation.

Although the adhesive layer does not stay fully exposed to cigarette smoke under clinical conditions, gaps may be present along the adhesive interface. Thus, in vitro study may possibly optimize this challenge, allowing for the analysis of the materials' behavior in extreme conditions. In addition, more efforts are needed in this area to understand (1) the impact of tobacco products on the physical properties of resin-based materials and also (2) the chemical interaction between volatile smoke compounds and these materials, including some factors such as the presence and type of the paper cigarette filters, frequency, and intensity of habit.

## 5. Conclusion

The water sorption and solubility values were significantly different among the adhesives tested, except for conventional systems. The cigarette smoke only significantly affected the water sorption and solubility of the self-etching adhesive system. Thus, the cigarette smoke can increase the water sorption and solubility of adhesive systems, promoting a decline in their physical properties and consequently a reduction in the service life of dental resin-bonds. 

## Figures and Tables

**Table 1:** The manufacturer's composition, classification, and batch number of adhesive systems used in this study.

Adhesive systems	Classification	Composition (wt% and # batch numbers)
Adper Scotchbond Multipurpose Adhesive (3 M/ESPE, St. Paul, MN, USA)	Conventional adhesive	BISGMA (60–70%); HEMA (30–40%), triphenylantimony (<0.5%) (no. 88940 and no. 22072).

Adper Scotchbond Multipurpose Primer (3 M/ESPE)	Conventional adhesive	HEMA (35–45%); water (40–50%); copolymer of acrylic and itaconic acids (10–20%) (no. 21785).

Adper Single Bond Plus Adhesive (3 M/ESPE)	Two-step etch-and-rinse	BISGMA (10–20%); HEMA (5–15%); UDMA (1–5%); ethyl alcohol (25–35%); glycerol 1,3 dimethacrylate (5–10%); copolymer of acrylic and itaconic acids (5–10%); water (<5% by wt); silane-treated silica (no. 67023BR).

Adper Easy One Adhesive (3 M/ESPE)	One-step self-etch	BISGMA (15–25%); HEMA (15–25%); ethanol (10–15%); water (10–15%); phosphoric acid-6-methacryloxy-hexylesters (5–15%); silane-treated silica (8–12%); 1,6-hexanediol dimethacrylate (5–10%); copolymer of acrylic and itaconic acid (1–5%); (dimethylamino)ethyl methacrylate (1–5%); CQ (1–3%); TPO (1–3%) (nos. 392306 and 404261).

Abbreviations: Bis-GMA: bisphenol A diglycidyl ether dimethacrylate; HEMA: 2-hydroxyethyl methacrylate; UDMA: diurethane dimethacrylate; CQ: camphorquinone; TPO: 2,4,6-trimethylbenzoyldiphenylphosphine oxide.

**Table 2:** Mean (standard deviations) of water sorption and solubility (*μ*g/mm^3^) of commercial adhesive systems tested in this study after immersion in deionized water and cigarette smoke during 21 days.

Adhesive systems	Water sorption		Solubility	
	Water	Cigarette smoke	Water	Cigarette smoke
Adper Scotchbond Multipurpose Adhesive	89.2 (4.0) Ca	89.3 (3.0) Ca	−10.8 (2.3) Ca	−10.1 (2.4) Ca
Adper Scotchbond Multipurpose Adhesive/Primer	87.1 (4.1) Ca	90.1 (2.1) Ca	−5.3 (2.0) Ca	−10.6 (1.8) Ca
Adper Single Bond Plus	268.5 (25.9) Aa	275.1 (8.0) Aa	83.3 (9.9) Ba	89.6 (7.2) Ba
Adper Easy One	216.5 (11.7) Bb	243.6 (9.2) Ba	113.2 (6.3) Ab	122.2 (4.6) Aa

Means followed by distinct letters represent statistically significant differences (2-way ANOVA/Tukey, *α* = 5%). Uppercased letters compare adhesives in the same column, and lowercased letters compare treatment in the same row for each sorption and solubility parameter.
